# The Mechanical Properties and Fracture Characteristics of Shale Layered Samples from the Lucaogou Formation Considering Natural Crack and Mineral Distribution

**DOI:** 10.3390/ma16175881

**Published:** 2023-08-28

**Authors:** Xiukuo Sun, Shouding Li, Xiao Li, Guanfang Li, Bo Zheng, Tianqiao Mao

**Affiliations:** 1 Key Laboratory of Shale Gas and Geoengineering, Institute of Geology and Geophysics, Chinese Academy of Sciences, Beijing 100029, China; sunxiukuo@mail.iggcas.ac.cn (X.S.); lixiao@mail.iggcas.ac.cn (X.L.); liguanfang@mail.iggcas.ac.cn (G.L.); zhengbo@mail.iggcas.ac.cn (B.Z.); maotianqiao@mail.iggcas.ac.cn (T.M.); 2 Innovation Academy of Earth Science, Chinese Academy of Sciences, Beijing 100029, China; 3 College of Earth and Planetary Sciences, University of Chinese Academy of Sciences, Beijing 100049, China

**Keywords:** Lucaogou Formation, real-time micro-CT scanning, mineral distribution, natural crack, fracture characteristics

## Abstract

Shale oil is one of the most promising alternative unconventional energies in the world, and recently the Lucaogou Formation showed significant exploration potential, becoming the primary target in northwestern China. This paper focuses on the mechanical properties and fracture characteristics of shale layered samples from the Lucaogou Formation, conducting uniaxial compressive tests with real-time micro-CT scanning, as well as mineral analysis after failure. It has been found that the mechanical and fracture features are both related to the composition, distribution, content and particle size of minerals, as well as natural fractures. The main crack tends to form in the weak mineral band, for example, calcite or clay band. Since the discontinuous stress usually forms at the interfaces of different minerals, the sample with several major minerals of close content is easier to break into a fractured zone, causing lower uniaxial compressive strength and elastic modulus, compared with the composition of only one dominant mineral. Also, the region will be more fractured after failure if the mineral particles there become smaller. Additionally, although natural cracks have a certain influence on the development of new fractures, not all of the natural ones will propagate into the final fracture network, some of them are just compacted and closed.

## 1. Introduction

Shale oil is one of the most important unconventional oil exploration targets [1,2], accounting for 20–50% of the total oil reserves across the world [1,3]. The principal method for exploring shale oil is large-scale volume fracturing. Unfortunately, only 20–50% of the fractured intervals are effective, while others are usually fail, thus contributing little to production [4,5]. Therefore, further research on the shale oil reservoirs is required, especially regarding the influential factors of crack development and mechanical properties.

In recent years, industrial shale oil has been explored in the Lucaogou Formation, Junggar Basin, China, which allows this formation to become the primary exploration target in the northwestern China. Particularly, the lithology of this shale oil reservoir represents great variation in a vertical direction with bedding planes [6], where the oil layers and interlayers are interbedded and distributed. This brings great difficulty to simulation of the reservoir. Therefore, it is fairly important to understand the mechanical and fracture properties of different layers, which is the key to optimizing a hydraulic fracturing strategy [7,8].

Many scholars have devoted their efforts to study of fracture mechanisms of reservoir rock, including the Lucaogou Formation. Zhang et al. [9] believed that during a uniaxial compression test, tensile cracks dominate overall, but more shear cracks are generated and developed at the final buckling failure. Zou et al. [10] found that the local heterogeneity in the continental shale formation can cause significant complexity and uncertainty in fractures. Li et al. [6] analyzed the crack height and its ability to penetrate bedding under the influence of layer thickness and lithology based on a triaxial hydraulic fracturing simulation. Zhang et al. [8] found that the bedding direction of a Lucaogou rock has a significant effect on its tensile strength and brittleness. Zou et al. [11] believed that the difference in rock mechanics between layers cannot effectively inhibit the growth of fracture height but has a significant effect on the fracture width. Wu et al. [12] found that the fracture is seriously disturbed by the bedding structure from the initiation stage.

The above studies on the fracture mechanism of rocks in the Lucaogou Formation are all in macro scale. The emergence and development of macroscopic cracks are essentially the aggregation and expansion of microscopic cracks. Therefore, the study of microfracture characteristics is also of great significance. However, such research focusing on the Lucaogou Formation is very rare. Research showed that the geomechanical characteristics of shales studied at the microscopic scale were often influenced by different minerals and/or porosity [13]. Padin et al. [14] discovered that the anisotropy of the fabric of the rock within the interface between clay and non-clay minerals that differ in size and strength and the orientation of the pre-existing microfracture networks are both significant parameters that dominate microfracture propagation mechanisms on organically rich rocks. Yang et al. [15] observed the crack closure, generation, growth, and penetration of Longmaxi shale. Wang et al. [16] observed the combined failure modes of tensile and shear from the fracture rose diagram of Longmaxi shale, and believed the formation and propagation of fractures were influenced by the layered structure and weak interlayer cementing medium.

Apart from observations of the fracture process in micro-scale, one of the most important factors that has a great influence on the rock strength and fracture mechanism is mineral composition and distribution. In previous research, the effect of minerals was mainly focused on composition. For example, the elastic modulus, Poisson’s ratio, the fracture pressure, the frictional strength, and critical porosity were all found to be correlated with the content of various minerals, such as quartz, carbonate, clay, or organic matter [17,18,19,20,21,22,23]. Pikryl et al. [24] thought that mineral grain size is the main microstructural factor controlling strength variation. It has also been found that cracks often generate along the borders of minerals for discontinuous stress and that the complexity of fractures is related to the organic matter content [21]. By converting the calcite on the limestone surface to smithsonite, Desouky et al. found that hardness, strength, modulus of elasticity, and permeability of the rock were changed, which meant that harder rock creates more fractures, increasing well productivity and ultimate recovery [25]. Nasseri et al. [26] believed that quantitative evaluation of mineral type, grain size, and shape, and tracking the micro-cracks would help to study the relationship between physical properties and mechanical properties such as the fracture toughness of rocks. However, the influence of mineral distribution has rarely been studied before.

Natural cracks also play an important role in volume fracturing, through the interaction between them and new cracks [27,28,29,30]. Gale et al. [31,32] found that in the bending test, the tensile strength of the sample with natural fractures is half that of the sample without natural fractures. Apart from that, there are also some experiments that observed the propagation law of new cracks on rock samples with artificial cracks [33]. However, the width of artificial cracks is often up to millimeter scale and significant stress concentration is easily formed at the tips, so the new cracks often create and propagate directly from here. The actual situation may be more complicated than that. Unfortunately, related experimental studies about the propagation of new fractures based on real natural cracks are rare.

To sum up, the current research on the fracture mechanism of shale layer samples in the Lucaogou Formation mainly focuses on the macro scale, lacking essential study of the micro scale. Also, there has been little research on the effects of mineral distribution and natural fractures. Therefore, in this paper, the fracture features and mechanical properties of shale layered samples from the Lucaogou Formation were investigated, based on uniaxial compressive tests with real-time micro-CT scanning. Considering that the resolution of CT imaging is up to 5 μm, which may expose some natural cracks in the sample, so the influence of natural cracks on new fractures was prepared to be studied if possible. After that, mineral analysis was conducted on the fractured samples with the ZEISS Merlin field emission scanning electron microscope (FE-SEM), and the crack patterns influenced by mineral distribution were analyzed.

## 2. Materials and Methods

### 2.1. Sample Preparation

**Figure 1:**
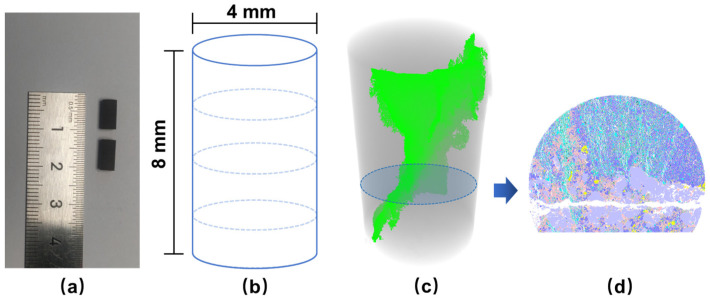
(**a**) Two samples from one rock block; (**b**) the direction of the bedding planes; (**c**) the illustration of the mineral analysis section (the green areas are cracks generated in the damaged sample); (**d**) the results of mineral analysis of the section in (**c**).

The shale layered samples for this experiment were obtained from the Lucaogou Formation in the Jimsar Sag Area along the southern margin of the Junggar Basin, China. Cylindrical samples were cored from 3 different rock blocks, using a hollow cylindrical, rotary core bit mounted on a drill press. Two specimens were taken from adjacent positions on each block. The top and bottom faces of these specimens were then cut and ground, to achieve flat and parallel surfaces for testing, with a precision of 60 µm. Finally, 6 samples of 4 mm in diameter and 8 mm in height were prepared. The prepared samples and the direction of the bedding planes are shown in Figure 1a,b. In particular, according to the logged results, the samples from Rock Blocks 1 and 3 belong to interlayers with relatively low oil content, while those for Rock Block 2 belong to an oil layer with higher oil content.

After the samples were prepared, the uniaxial compressive tests and mineral analysis were proceeded in turn. This means that the mineral analysis was conducted on fractured samples. According to the fracture pattern, a section parallel to the end face with a relatively large area was selected (Figure 1c,d). Mechanical polishing and argonionmilling were then carried out on the cut section of the specimen for the next mineral analysis.

### 2.2. Testing Devices

As described before, the experiments in this research contain two stages: The first stage is the uniaxial compression test with real-time micro-CT scanning, aiming at studying the progressive fracture features and mechanical properties of shale layered samples. The second stage is the mineral analysis, focusing on the spatial relationship between the cracks and the mineral distribution. This means two advanced experiment systems were conducted and both of them were place at the Institute of Geology and Geophysics, Chinese Academy of Sciences.

**Figure 2:**
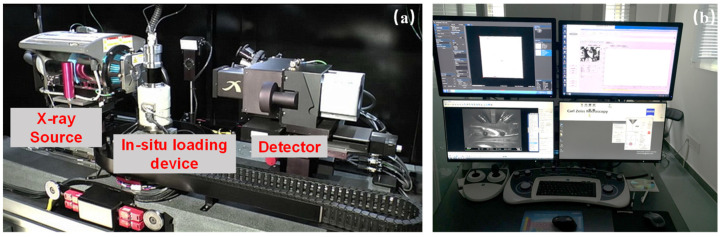
(**a**) The ZEISS Xradia 520 Versa 3-D X-ray microscope (micro-CT); (**b**) the ZEISS Merlin field emission scanning electron microscope (FE-SEM).

The CT equipment used in this test is a ZEISS Xradia Versa 520 3D X-ray microscope, commonly known as micro-CT. Different from the traditional X-ray CT, this micro-CT has a microscope-level magnification architecture that combines geometric and optical magnification [14]. The main components of the micro-CT are an X-ray source, an in situ loading device, and a detector (Figure 2a).The energy of the X-ray source ranges from 30 to 160 kV and the highest imaging resolution can reach 0.7 μm, which is the highest among similar instruments at the micron level. The internally equipped in situ uniaxial compressor (Deben MICROTEST compression stage) collects stress and strain values at a rate of 3–10/s, with a maximum loading pressure of 5000 N and a loading rate of 0.03 mm/min. This loading apparatus was placed on the rotary table of the CT facility, so that it could rotate together. Therefore, the real-time CT scanning without unloading could be achieved by this micro-CT. In this research, the resolution of CT images is 5 μm/pixel.

The equipment used to analyze the mineral composition and distribution is the ZEISS Merlin field emission scanning electron microscope (FE-SEM, Figure 2b). This facility is equipped with the AMICS automatic large-area mineral analysis system. The smallest identification area of mineral particles in AMICS is up to 0.5 μm^2^. In this research, the resolution of the mineral analysis image is 0.586 μm/pixel.

### 2.3. Experiment Procedures

After the 6 cylindrical specimens (4 mm in diameter and 8 mm in height) were prepared, the uniaxial compression test on the micro-CT was conducted. During this loading process, temporary pauses were required for CT scanning. To determine the appropriate stress for the scan, the strength and failure process were learned roughly in advance by performing conventional uniaxial compression experiments without CT on Samples 1-1, 2-1, and 3-1. Afterwards, the same tests were conducted on Samples 1-2, 2-2, and 3-2 with real-time CT scanning. The stress–strain curves and scanning points are shown in Section 3.2.

Another important aim for this research was to study the crack propagation under the influence of mineral distribution. Therefore, after the CT tests, according to the fracture pattern of each sample, a section parallel to the end face with a relatively large area was selected (Figure 1c,d), then the mineral analysis was conducted on this section by the AMICS system on FE-SEM (Figure 2b).

**Figure 3:**
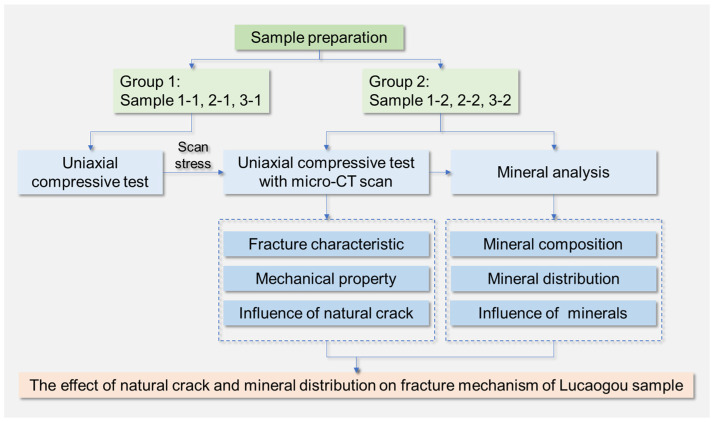
The flow chart of the paper organization.

The flow chart of the organization of this paper is shown in Figure 3.

## 3. Results and Discussion

### 3.1. Mineral Composition

**Figure 4:**
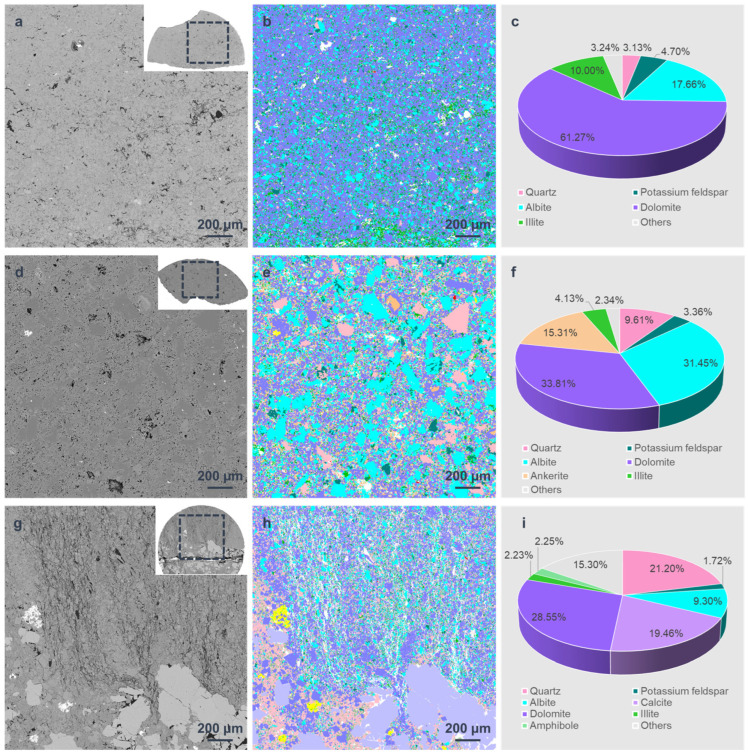
The mineral analysis for the three samples. (**a**) The backscatter selective detector (BSD) image of Sample 1-2; (**b**) the AMICS result of mineral composition mapping of Sample 1-2; (**c**) the mineralogical composition of Sample 1-2. (**d**–**f**) Are the same figures for Sample 2-2; (**g**–**i**) are the same figures for Sample 3-2.

The mineral analysis was conducted using the AMICS system on FE-SEM after sample failure. In order to acquire as much information as possible about the spatial distribution of fractures and minerals, the region next to the main crack was tested. This is why the tested areas of the three samples are irregular in shape (the small pattern in Figure 4a,d,g). The main mineral in all these samples is dolomite, with a content of 61.27%, 33.81%, and 28.55%, respectively. Another main mineral composition is albite, which has the highest content of 31.45% in Sample 2-2, almost reaching the content of dolomite. The quartz content is greatly varied; it is 3.13% and 9.61% in Samples 1-2 and 2-2, while up to 21.20% in Sample 3-2. Another difference is that only Sample 2-2 contains ankerite with a relatively high content of 15.31%, and Sample 3-2 contains a small amount of amphibole. In particular, the special feature of sample 3-2 is that it contains a calcite band, which enables the calcite content to be as high as 19.46%. Apart from that, the clay content of Samples 2-2 and 3-2 is similar (4.13% and 2.23%), less than that of Sample 1-2 (10.00%).

The mineral distribution of the three samples shows different characteristics. For Sample 1-2, the dolomite content is more than 60%, which could be considered the “dominant mineral”. It is like the “matrix” composition here, with other mineral particles scattered inside, such as quartz, feldspar, and illite. Among them, the content of albite and illite is more than 10%, respectively, thought of as the “major mineral”. However, in Sample 2-2, the mineral distribution features are different. Given the similar content of dolomite and albite, the particles of these two minerals are mixed and scattered together, along with quartz, ankerite, and others. For Sample 3-2, there is a calcite band. Small dolomite, quartz, and albite particles are also mixed and scattered in other parts of the section. These different distribution features of minerals have an influence on the mechanical and fracture properties of the samples.

### 3.2. The Progressive Fracture Characteristic and Mechanical Property

**Figure 5:**
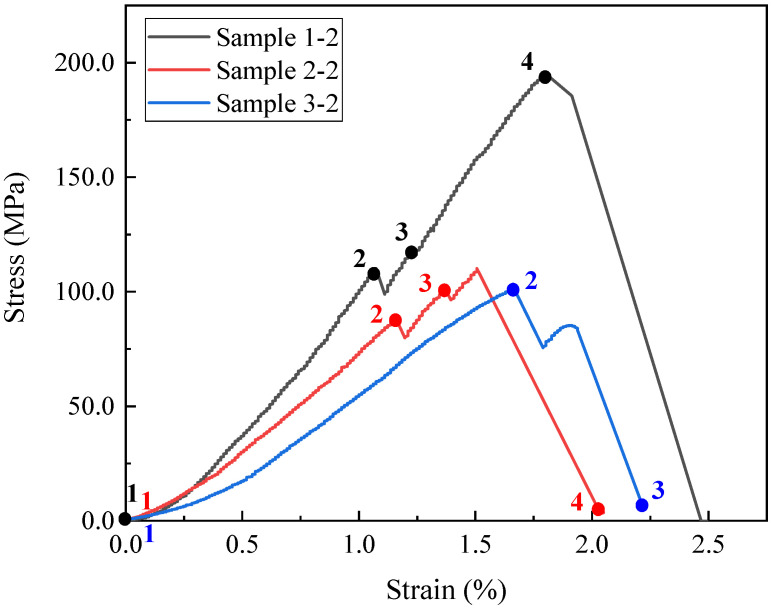
The stress–strain curves for three samples with scanning step numbers.

**Figure 6:**
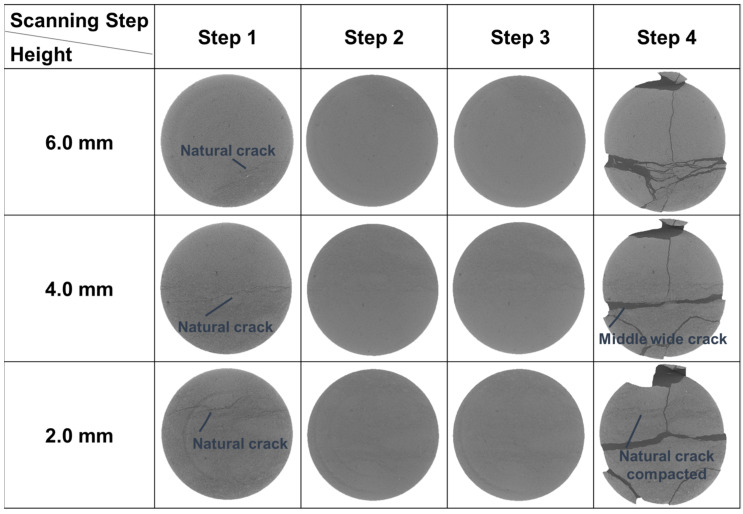
The CT images of Sample 1-2 at different heights in different scanting steps.

**Figure 7:**
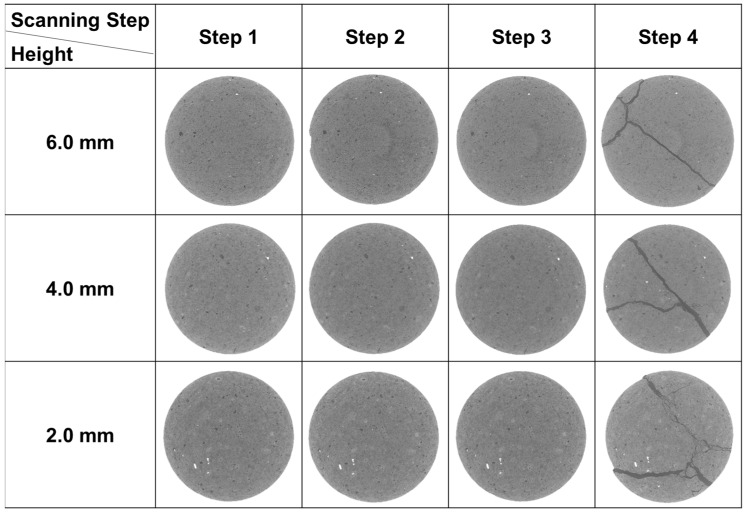
The CT images of Sample 2-2 at different heights in different scanting steps.

**Figure 8:**
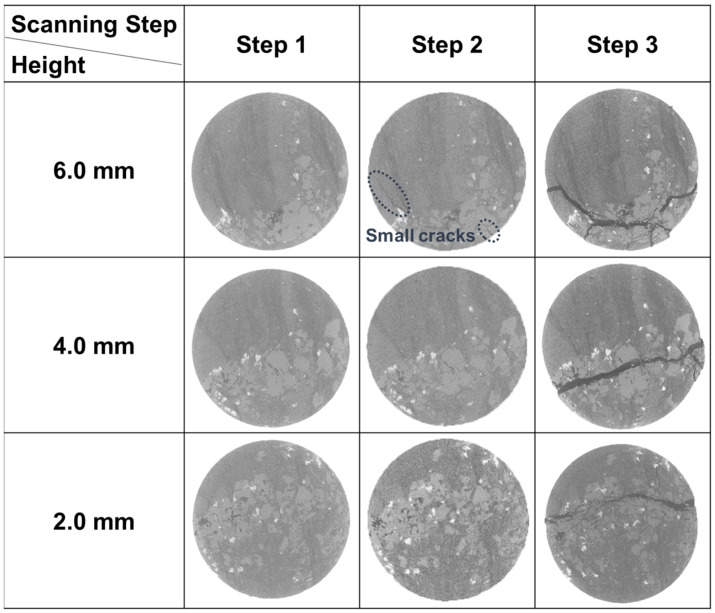
The CT images of Sample 3-2 at different heights in different scanting steps.

After the uniaxial compression tests with real-time micro-CT scanning, the stress–strain curves of the three samples were obtained (Figure 5). The points with numbers on each curve show the scanning steps in the experiments. Among them, Samples 1-2 and 2-2 experienced 4 scans, and Sample 3-2 experienced 3 scans. The CT images at different heights of the samples are shown in Figure 6, Figure 7 and Figure 8. From these images, the progressive fracture processes could be analyzed.

There are some natural cracks in Sample 1-2 according to the initial structure in scanning Step 1 in Figure 6. After the loading before Step 3, these cracks were compacted and closed, which could be clearly observed in this figure (Steps 2, 3). The new fractures were not observed until the stress reached peak value, and such phenomena also appeared in Sample 2-2 (Figure 7). For Samples 1-2 and 2-2, considering that the resolution of CT images is 5μm/pixel, it could be inferred that the new cracks whose width were larger than 5 μm initiated after Step 3 when the stress level was 58.59% and 86.09%, respectively. The stress level here equals the ratio of the current stress to the peak stress. In other words, during uniaxial compression, new cracks wider than 5 μm would not develop until the stress level was higher than 86.09%.

As for Sample 3-2, the small cracks had already been initiated in scanning Step 2 (Figure 8), when the stress reached peak value. After this scan, the loading continued, at the same time the cracks kept developing and the stress was increased by 9.30 MPa. Then the whole sample fractured. This means that once the cracks are initiated, the sample would fail soon.

The mechanical properties were also investigated. The surface porosity, uniaxial compressive strength (UCS), and elasticity modulus (E) are shown in Table 1. The surface porosity of Sample 2-2 is up to 13.50%, while that of Samples 1-2 and 3-2 is only 5.07% and 10.30%, respectively, less than the value in Sample 2-2. This is because Sample 2-2 belongs to an oil layer with a relatively high shale oil content and the other two samples are both from interlayers. As for the UCS, it is 198.45 MPa for Sample 1-2, 1.72 times higher than that of Sample 2-2, although there are natural cracks in the first sample (Figure 6). Also, the E of Sample 1-2 is greater. Apart from the effect of lower surface porosity, the different distributions of minerals also contribute to this variation. The dolomite and albite in Sample 2-2 have similar content of more than 30% and the particles of these two minerals are mixed and scattered. This results in discontinuous stress at the interfaces, which reduces the ability to withstand external forces or resist deformation, leading to lower UCS and E values. In addition, the UCS and E are even lower in Sample 3-2. This is also related to the mineral distribution, in this case, the calcite band. This band passes obliquely through the sample. Since calcite is weak, with a lower Moh’s hardness than other major minerals, the main crack tends to generate in it more easily. Therefore, the UCS and E here are the lowest among the three samples.

### 3.3. Effects of Mineral Distribution on Crack Propagation

**Figure 9:**
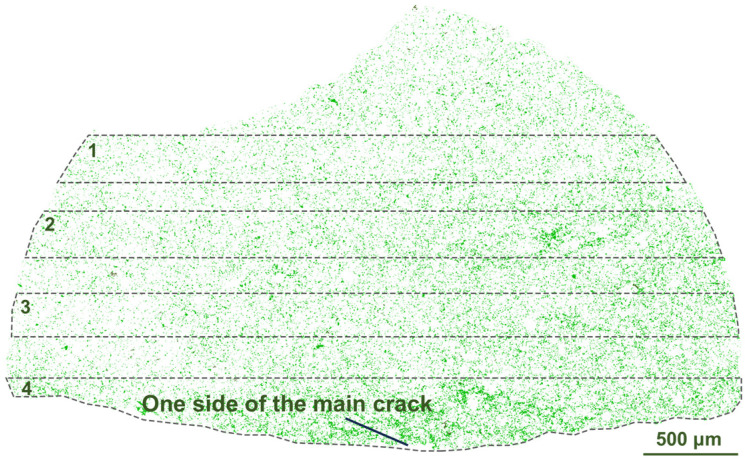
The distribution of clay minerals (including illite and chlorite) in Sample 1-2.

After the failure of the samples, mineral analysis was conducted based on the AMICS system on FE-SEM to study the influence of mineral distribution on crack propagation. Since the samples were fractured, the tested sections were all irregular in shape. According to the mineral analysis results, the crack development is closely related to mineral distribution. For Sample 1-2, the dominant mineral is dolomite and other minerals, such as quartz, feldspar and illite, are all scattered throughout it (Figure 4b). Among them, it seems that next to the main crack, some clay minerals are concentrated. In order to verify that quantitatively, the clay, including illite and chlorite, was extracted alone in Figure 9. The content of clay mineral in Regions 1–4 is 4.87%, 7.60%, 6.98%, and 14.61%, respectively. Apparently, it is the highest in Region 4, which is right next to the main crack. Considering that Moh’s hardness is 1-2 for clay, the lowest among all the minerals in Sample 1-2 (for example, it is 7 for quartz, 6–6.5 for feldspar, and 3–3.5 for dolomite), discontinuous stress is easily formed at the interfaces between clay and other minerals. This leads to the formation of the main fracture in the band where clay minerals gather.

**Figure 10:**
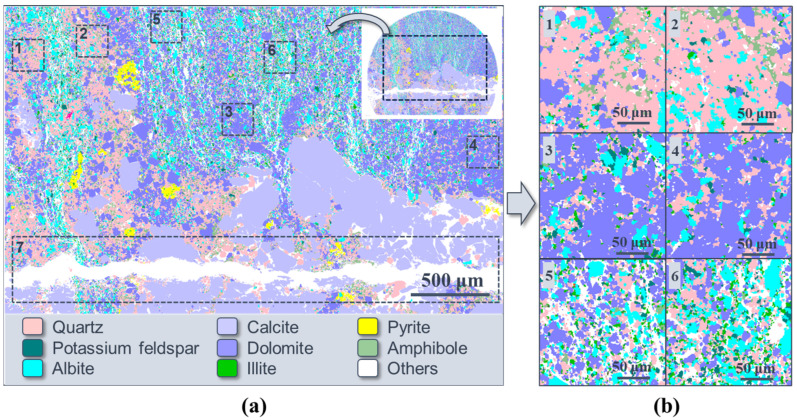
(**a**) The distributions of minerals and cracks in Sample 3-2; (**b**) the enlarged visions of Regions 1–6 in (**a**).

A similar phenomenon could be observed in Sample 3-2 (Figure 10a). It is clearly shown that there is a calcite band in this sample and the main crack developed right in it (Figure 8 and Figure 10a). The white color in Figure 10 represents cracks. In Region 7, not counting the area of the main crack, the content of calcite is as high as 55.04%. Additionally, Moh’s hardness for calcite is 3, which is also relatively low compared with other major minerals (quartz, feldspar, and dolomite), so the calcite band is actually a weak band here. Therefore, the main crack finally propagated in this band.

Apart from the main crack, there are also large fractured zones in Sample 3-2 (Figure 10). Like the main crack, the generation of fractured zones was also related to the mineral composition and distribution, and Regions 1–6 in Figure 10 were chosen to illustrate this. These regions share the same shape and size, and their mineral content is shown in Table 2. In the first two regions (Regions 1 and 2), the quartz content is the highest, with an average of 64.66%, and it is much higher than other minerals. The situation in Regions 3 and 4 is similar, the difference is that the highest mineral by content is dolomite, reaching an average of 66.25%. In these four regions, the content of fractures is only 3–6%. However, things are different in Regions 5 and 6. The content of three major minerals here, quartz, albite and dolomite, is from 10% to 30%, relatively closer compared with the first four regions. The total content of the three minerals accounts for about 60% in total for each region (Regions 5 and 6), and the fracture ratio is up to an average of 25.17%, a lot more than that of the other four regions. This means that Regions 5 and 6 are fractured zones, and here we consider a fractured zone to be a zone where the area ratio of dispersed fractures is more than 20%. Therefore, apart from the weak band, like the clay or calcite band in this experiment, if the region is dominated by one kind of hard mineral (such as quartz or dolomite), it tends not to generate small cracks here; while if there are more than one major minerals with similar content, this region can easily become a fractured zone.

**Figure 11:**
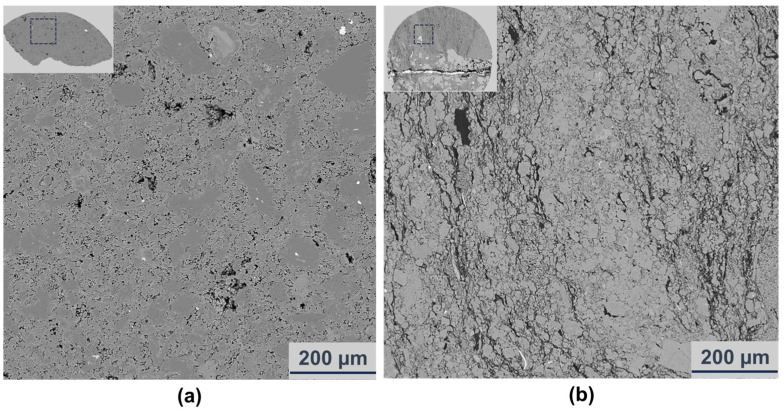
The backscatter selective detector (BSD) image of Samples 2-2 (**a**) and 3-2 (**b**).

However, unlike Sample 3-2, although the content of two major minerals, albite and dolomite, is also close in Sample 2-2 (31.45% and 33.81%, Figure 4), the fracture zone with small cracks does not form here. This phenomenon is shown in Figure 11. From the comparison of two backscatter selective detector (BSD) images, it is completely clear that there are few small cracks in Sample 2-2 (Figure 11a), while Sample 3-2 has already fractured with dense thin cracks (Figure 11b). Why is the phenomenon different? It is supposed that this is related to the size of the mineral particles. As can be seen from Figure 4, one of the most obvious differences between the two samples is the size of the albite particles. Thus, the area ratio of albite particles of different sizes to all albite particles was calculated in Table 3. The long axis of each particle was considered to be its size. For Sample 2-2, 38.16% of the albite particles are smaller than 50 μm, but for Sample 3-2, the content is 77.97%, more than twice as much as that of Sample 2-2. This means that 61.84% and 22.03% of albite particles, respectively, are larger than 50μm for the two specimens. Among them, in Sample 2-2, 45.36% of particles are larger than 100 μm, while for none in Sample 3-2 is this the case. This result illustrates that most of the albite particles in Sample 2-2 are relatively larger than those in Sample 3-2, which reduces the contact face between albite and other minerals greatly. Considering that the discontinuous stress at interfaces between different minerals promote the generation of cracks, it is reasonable that the small cracks were limited in Sample 2-2. Apart from that, the difference in fracture development between the two samples may also be related to other factors (e.g., cementation), but this is outside the scope of this article.

### 3.4. The Influence of Natural Cracks on Final Cracks

**Figure 12:**
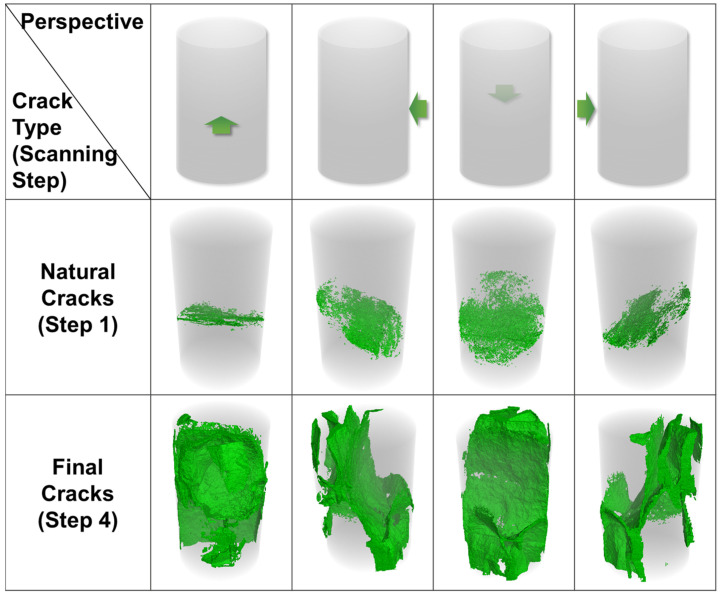
The natural and final cracks from four different perspectives in Sample 1-2.

According to the micro-CT images, the natural cracks that existed in Sample 1-2 are shown in Figure 6 (Step 1). With an image resolution of 5 μm/pixel, the width of the natural cracks is 5–15 μm. During the loading process, these cracks were compacted and closed at scanning Steps 2 and 3, at stress levels of 51.52% and 58.59% (Figure 6). After the stress arrived at the peak value, the final crack network was formed in the sample. It turns out that the influence of natural cracks on final fractures is different. The final fractures at a height of 6.0 mm developed along the original cracks, but those at a height of 2.0 mm did not completely (Figure 6). As for the fractures at a height of 4.0 mm, the left part of the middle wide fracture propagated along the natural one, while the right part did not. After the reconstruction of the CT images, it was found that these natural cracks in different slices belong to one three-dimensional crack (Figure 12). This original crack traversed through 453 CT slices in all before loading, and the final cracks propagated along the natural ones in 179 slices (39.51% of the total natural crack), partly propagated along it in 139 slices (30.68%), while in 135 slices the final cracks totally developed not along the original ones (29.80%). The final cracks are also shown in Figure 12. Therefore, although natural cracks have a certain influence on the generation of final fractures, the final ones do not necessarily extend completely along the original ones. Some natural cracks are just compacted and closed (Figure 6, at the height of 2.0 mm). This shows that the propagation of cracks is the result of the comprehensive action of many factors and the natural fracture is only one of them, which cannot totally decide where the cracks develop.

## 4. Conclusions

In this study, the fracture characteristics and mechanical properties of shale layered samples from the Lucaogou Formation were investigated, according to the uniaxial compression tests with real-time micro-CT scanning, and mineral analysis by the AMICS system on FE-SEM. It was found that the composition, distribution, content, and particle size of minerals, as well as natural cracks, are all related to the mechanical and fracture features. The specific conclusions are as follows:

(1) The UCS and E are affected by mineral composition and distribution. If there is more than one major mineral with close content in the sample, the discontinuous stress will appear at their interfaces, resulting in fractures. Thus, the UCS and E of such samples are relatively low, compared with a composition with only one dominant mineral.
(2) The main crack is easy to form in the weak mineral band (e.g., calcite and clay); a fractured zone with thin cracks tends to form when several different minerals close in content are mixed and scattered, and the region will be more fractured after failure if the mineral particles there become smaller.
(3) The development of cracks is the comprehensive result of many factors and a natural crack is one of these. Most natural cracks propagate to formed a final fracture network but there were still more than 30% of them which were just compacted, not contributing to new cracks.

## Figures and Tables

**Table 1:** The mechanical parameters of the three samples.

Sample Number	Oil Layer/Interlayer	Surface Porosity (%)	UCS (MPa)	E (GPa)
1-2	Interlayer	5.07	198.45	12.66
2-2	Oil layer	13.50	115.32	8.67
3-2	Interlayer	10.30	102.95	7.75

**Table 2:** The content of minerals of Regions 1–6 in Sample 3-2 (%).

Region Number	1	2	3	4	5	6
Quartz	64.00	65.31	21.56	17.25	16.56	20.23
Albite	10.12	9.25	5.86	6.58	13.45	22.52
Potassium feldspar	0.77	1.48	1.28	1.00	4.04	4.80
Dolomite	12.21	10.14	63.32	69.17	29.21	22.54
Amphibole	7.76	8.02	0.51	0.69	1.28	0.79
Clay	0.71	0.96	1.08	1.61	5.06	7.33
Crack and voids	3.65	4.39	5.76	3.25	29.65	20.68

**Table 3:** The area ratio of albite particles of different sizes to all albite particles.

Particle Size Range (μm)	Sample 2-2 (%)	Sample 3-2 (%)
1–10	8.36	17.78
10–20	12.00	25.84
20–30	6.25	17.14
30–40	7.61	9.82
40–50	3.93	7.39
50–100	16.48	22.03
>100	45.36	0.00
All	100	100

## Data Availability

All the relevant data are available in the manuscript.
